# Participants Characteristics of a Park-Based Physical Activity Intervention in an Urban Context—A Cross-Sectional Study in Bologna, Italy

**DOI:** 10.3390/healthcare11162287

**Published:** 2023-08-14

**Authors:** Alessandro Bianconi, Alice Masini, Francesco Sanmarchi, Stefania Toselli, Alessia Grigoletto, Sofia Marini, Giuseppe Barone, Erika Pinelli, Raffaele Zinno, Mario Mauro, Pietro Loro Pilone, Sonia Arduini, Mauro Vitiello, Bruno Vicentini, Giorgia Boldrini, Muriel Assunta Musti, Paolo Pandolfi, Maurizio Liberti, Gerardo Astorino, Pasqualino Maietta Latessa, Laura Bragonzoni, Laura Dallolio

**Affiliations:** 1 Department of Biomedical and Neuromotor Sciences, University of Bologna, 40126 Bologna, Italy; alessandro.bianconi4@studio.unibo.it (A.B.); alice.masini7@unibo.it (A.M.); stefania.toselli@unibo.it (S.T.); alessia.grigoletto2@unibo.it (A.G.); laura.dallolio@unibo.it (L.D.); 2 Department of Life Quality Studies, University of Bologna, 47921 Rimini, Italy; sofia.marini2@unibo.it (S.M.); giuseppe.barone8@unibo.it (G.B.); erika.pinelli2@unibo.it (E.P.); raffaele.zinno2@unibo.it (R.Z.); mario.mauro4@unibo.it (M.M.); pasqualino.maietta@unibo.it (P.M.L.); laura.bragonzoni4@unibo.it (L.B.); 3 Department of Public Health, AUSL di Bologna, 40124 Bologna, Italy; p.loropilone@ausl.bologna.it (P.L.P.); sonia.arduini@auslromagna.it (S.A.); mauro.vitiello@auslromagna.it (M.V.); murielassunta.musti@ausl.bologna.it (M.A.M.); paolo.pandolfi@ausl.bologna.it (P.P.); maurizio.liberti@ausl.bologna.it (M.L.); gerardo.astorino@ausl.bologna.it (G.A.); 4 Culture and City Promotion Department, Municipality of Bologna, 40126 Bologna, Italy; bruno.vicentini@comune.bologna.it (B.V.); giorgia.boldrini@comune.bologna.it (G.B.)

**Keywords:** physical activity, urban health, community health promotion, exercise, community-based intervention

## Abstract

Physical inactivity, a leading risk factor for chronic diseases and premature death, is prevalent worldwide. This study focuses on participant profiles and factors influencing continued participation and physical activity (PA) levels in the “Moving Parks” PA intervention in Bologna, Italy. This intervention offers free group activities in city parks during the summer. A questionnaire was administered to participants in 2022, gathering data on socio-demographic information, chronic diseases, participation in previous iterations, and PA levels. Out of 596 respondents, 85% were women, and the average age was 50. About 56% held a university degree, and 73% exercised regularly in the winter. Continuous participation was linked to older age, higher education, and chronic conditions. Prior participation predicted higher winter PA levels. Notably, the majority of participants were educated, physically active women. While the project continually engages individuals with higher education and chronic conditions, it needs further tailoring to reach less represented populations.

## 1. Introduction

Physical activity (PA) is an essential element for human health, and it confers numerous benefits that contribute to reducing all-cause and cardiovascular-specific mortality, reducing the incidence of chronic conditions such as hypertension, obesity, and type-2 diabetes, and achieving better mental health outcomes [1]. Conversely, physical inactivity, defined as failing to meet PA levels recommended by the World Health Organization (WHO) guidelines, is associated with several health problems and contributes to the global burden of non-communicable diseases [2,3].

To achieve substantial health benefits, the WHO recommends doing at least 150–300 min of moderate-intensity aerobic physical activity (MPA) or at least 75–150 min of vigorous-intensity aerobic physical activity (VPA) or an equivalent combination of MPA and VPA per week for adults [1]. Unfortunately, more than 25% of the world’s population does not comply with these recommendations [4]. In Italy, only 48% of adults aged between 18 and 69 met sufficient PA levels in 2021 [5].

Urban living is linked to physically inactive lifestyles [6], and the rate at which the world population lives in cities is expected to grow during the next few decades, with more than two people in every three expected to live in urban areas by 2050 [7]. Many aspects of urban living have been shown to be related to lower levels of PA and higher rates of sedentary behavior [2,8].

However, some aspects of the built environment, such as urban greenness, may nudge residents’ PA levels [9,10]. Particularly, the presence of residential public Urban Green Space (UGS) has been associated with higher rates of PA among citizens living nearby [11]. Studies have shown that individuals who live in proximity to parks or other UGS tend to be more likely to engage in PA (i.e., walking or cycling) [12,13]. Conceivably, UGS may provide a more attractive and appealing setting for PA compared to other urban environments, which may be perceived as unattractive [14]. Moreover, UGS has been shown to be associated with improved mental well-being [15] and reduced levels of air pollutants and ambient temperature during the summer [16,17], representing a decreased exposure to known PA barriers [18,19]. Additionally, the effects of UGS on PA levels have been proposed as one of the explanatory mechanisms of the association between urban greenness and better health in individuals living near those spaces [20,21].

In recent years, numerous PA promotion interventions have tried to leverage the benefits of UGS with the aim of increasing PA levels in urban populations. These interventions may consist of improving UGS, such as by enhancing the presence of sport facilities or the creation of trail paths, increasing community engagement, or implementing group physical activities or group exercise sessions in public green areas [22,23,24].

Group outdoor-based PA promotion programs may be associated with increased PA levels [24]. These programs, typically involving organized group exercise sessions or lessons in outdoor environments, may provide a supportive and social context for PA, increasing motivation and adherence to an active lifestyle [25]. Practicing exercises in an outdoor environment has multiple positive effects, such as sustaining physical health [26] and enhancing mental health [27]. As a matter of fact, a growing body of evidence suggests that practicing physical exercise in an outdoor environment can produce greater physical and mental health benefits than exercise in other settings [28,29]. In 2010, the Municipality of Bologna (Italy), in collaboration with the Department of Public Health and local sport clubs, launched the “Moving Parks” project. This project aimed to promote free group PAs in public parks during the spring and summer among citizens in order to improve their health-related quality of life.

To our knowledge, “Moving Parks” is one of the most important outdoor-based PA promotion initiatives based in Northern Italy. The added value of this project lies in the involvement of professional figures that work closely with stakeholders in order to offer a real, structured, and sustainable initiative to the community.

The “Moving Parks” project has already been successfully implemented in the last 10 years. Adults and older adults who have attended the project have obtained important benefits in terms of both physical and psychological well-being [30,31]. However, little is known about how initiatives like “Moving Parks” are used by the general population. For this reason, the aim of the present study was to assess the user profile of the “Moving Parks” project and to analyze the determinants of PA and of continuous annual involvement in the project among participants. The results of this study will be useful to improve the implementation phase of the project by reaching population categories that could benefit most from it.

## 2. Materials and Methods

### 2.1. Study Design and Participant

The “Moving Parks” project is an ongoing initiative that takes place during the summer months in the majority of public parks in Bologna, a city in Northeastern Italy with a population of 400,000 inhabitants. Public green areas, which encompass 10 km^2^ of the municipal region, include 6 km^2^ of 250 public parks and gardens. The project was initiated in 2010 by the Municipality of Bologna and the Local Health Authority to encourage outdoor physical activity, and it comprises various forms of group activities (i.e., yoga, green volley, Nordic Walking, and capoeira). Each activity is offered freely from Monday to Friday and administered by certified instructors from various sports associations. In order to participate, citizens may access the activity of interest directly at the park or reserve a spot for those activities with a limited number of participants. The initiative is advertised through a wide range of media, including street billboards, the LHA and municipal websites, and social media. The activities take place in one selected public park for each district of the city in order to guarantee accessibility to all citizens.

This cross-sectional study analyzed data collected during the 2022 edition of the initiative, where seven parks were selected to serve as venues for the project. The study was designed based on the Strengthening the Reporting of Observational Studies in Epidemiology (STROBE) reporting guidelines [32]. The University of Bologna Bioethics Committee granted approval for the study (Prot. No. N 169182).

### 2.2. Instruments

A questionnaire was administered voluntarily at the beginning of the intervention period (June 2022) and at the end of the activity sessions in every activity location. The target population of this study coincided with the participants of the project. The data was collected anonymously by the research team. All participants were adults and provided informed consent for data processing. The English version of the questionnaire can be found in the Supplementary Material.

Socio-demographic information, including age, gender, and educational level, was gathered from participants, along with smoking habits, the presence of chronic diseases, participation in previous editions, and PA habits.

The self-reported presence of PA practice during the winter months before the beginning of the “Moving Parks” project was used to evaluate physical activity habits. The weekly hours spent on Moderate Physical Activity (MPA) and Vigorous Physical Activity (VPA) were transformed into a dichotomous variable based on the WHO’s PA recommendations for adults as cut-offs to determine whether the participants had sufficient MPA or VPA levels. The recommended PA variable was computed to identify individuals who have adequate levels of MPA or VPA.

### 2.3. Data Analysis

Participants’ characteristics and responses were summarized using mean and standard deviation and absolute and relative frequencies, as appropriate. Missing values were managed as “Missing At Random”.

A logistic regression model was employed to analyze the determinants of PA habits in the study sample. Each model was adjusted for gender, age, educational level, smoking habit, and the presence of chronic health conditions. The logistic regression model’s assumptions (i.e., independence of observations, absence of multicollinearity, sufficient sample size, and appropriateness of the logit function) were validated to ensure the reliability of our analysis. Moreover, participation in previous editions of “Moving Parks” was considered a possible covariate to evaluate the effects of this intervention on participants’ PA levels.

Secondary analysis investigated the predictive factors of annual continuous participation in the “Moving Parks” project using multiple regression models adjusted for gender, age, educational level, smoking habit, and the presence of chronic health conditions.

Lastly, the associations between socio-demographic and lifestyle variables (gender, age, educational level, smoking habit, and presence of chronic health conditions) and the type of media through which each participant became aware of the project were analyzed using a multiple regression model. Each type of medium was analyzed as an ad hoc dummy variable.

The statistical significance level was set at *p* < 0.05. All analyses were performed using R-Studio statistical software (RStudio, PBC, Boston, MA, USA) [33].

## 3. Results

### 3.1. Population Characteristics

Data from 596 participants was collected for the purpose of the study. Of which, 503 (85%) were female and 91 (15%) were male. The mean age of the participants was 50 (SD = 16). A total of 61 (10%) had an educational level lower than high school, 201 (34%) had a high school degree, and 331 (56%) had a university degree. Out of the entire sample, 438 participants (73%) reported exercising regularly during the winter months. Of those already active during the winter months, 237 (54%) reported meeting the WHO-recommended MPA levels and 170 (41%) the VPA ones. Thus, of the participants, 290 (49%) reported achieving the WHO recommendation for adequate PA levels.

The main source thanks to which each participant became aware of the project was word-of-mouth (n = 201, 35%), followed by the internet (n = 133, 23%). Moreover, other sources included social media (n = 88, 15%), advertisements (n = 34, 5.9%), newsletters (n = 31, 5.3%), seeing the activities in local parks (n = 26, 4.5%), and television (n = 1, 0.2%).

When asked about the reasons for participating in the project, 147 individuals (25%) reported that they felt good after participating, 146 (25%) reported that the project allowed them to be outdoors and in nature, 132 (23%) reported that they appreciated the activities, 67 (12%) reported that it was free, 47 (8.1%) reported that the parks were easy and convenient to reach, 13 (2.2%) reported that they appreciated other participants presence, and 26 (4.5%) reported that a mix of the above reasons applied.

A total of 312 participants (52%) reported that they took part in previous editions of the “Moving Parks” project. The results of the descriptive analysis are summarized in Table 1.

### 3.2. Logistic Regression Analyses

The results of the logistic regression analysis are summarized in Table 2. Age, educational level, and having a chronic health condition were found to be predictive factors of continuous annual participation in the project. In particular, older age was associated with higher odds of being a participant in previous editions of the project (OR = 1.04 [+4%], 95% CI = 1.03–1.05, *p* < 0.001). The likelihood of continuous participation was higher in those having a high school degree (OR = 2.01 [+101%], 95% CI = 1.03–3.92, *p* = 0.041) or having at least a university degree (OR = 2.05 [+105%], 95% CI = 1.04–4.06, *p* = 0.039), compared with those having an educational level lower than high school. Having a chronic disease resulted in a higher likelihood of continuous participation in the project (OR = 1.70 [+70%], 95% CI = 1.08–2.71, *p* = 0.023).

Continuous participation in previous editions of the project was associated with higher PA levels during the winter months (OR = 1.82 [+82%], 95% CI = 1.21–2.76, *p* = 0.004). Moreover, continuous participation was also associated with higher recommended PA levels during the winter months (OR = 1.46 [+46%], 95% CI = 1.02–2.08, *p* = 0.038). Results for these analyses are reported in Table 3.

Multiple logistic regression analyses performed for each type of dissemination medium highlighted an existing association between older age and becoming aware of the project through participants’ own sport associations. Moreover, having a high school or university degree was associated with lower odds of becoming aware of the project through word-of-mouth, compared with having less than a high school degree. Female gender and lower age results were associated with becoming aware of the project through social networks. Male gender and having a chronic condition resulted in predictive factors for becoming aware of the project through advertising flyers or billboards.

Complete results are reported in the Supplementary Materials (Table S1).

## 4. Discussion

### 4.1. Summary of the Results

This cross-sectional study analyzed the characteristics of the users of the “Moving Parks” project, the participants’ sociodemographic and lifestyle factors linked to their continuous participation in the project, and how different population subgroups became aware of the project.

Our results revealed that the majority of participants in the 2022 edition of the “Moving Parks” project were women and individuals with an educational level equal to a university degree. The mean age of the sample was comparable to that of the general population of the target municipality, which was the target population [33]. However, further analysis of gender and education distribution revealed a disparity in participation rates that favored women and those with higher education levels [34]. These results suggest the existence of barriers that hindered the participation of underrepresented subpopulations in the project.

Previous research has indicated that individuals with higher levels of education are more likely to engage in health promotion activities, including park-based PA interventions [35]. This may be due to several factors, such as higher levels of PA among those with higher education levels and greater access to information and resources related to health promotion [36]. Furthermore, these individuals may also have greater personal and financial resources to participate in such activities and an increased sense of self-efficacy and motivation to engage in behaviors that promote health and wellbeing [37].

The perception of the environment changes based on gender [38]. In particular, UGS are subject to gender-based differences in usage patterns. A research study revealed that women have lower access rates to public UGS compared to men [39]. Furthermore, self-perceived social safety may impact outdoor physical activities among women [40]. Factors such as visibility, cleanliness, and the presence of amenities have a more pronounced effect on women’s perceived safety compared to men’s, suggesting the important role of aspects like UGS design in mediating gender-based differences in public green space utilization [40]. While the gender distribution of participants in the “Moving Parks” project was unequal, the higher participation of women suggests that group-based PA interventions may nudge a more gender-inclusive use of public parks, which aligns with multiple goals of the UN Agenda 2030 for Sustainable Development [41].

Individuals that participated in the “Moving Parks” project had higher average levels of PA compared to the target population [42]. Studies have found that individuals participating in PA promotion interventions tend to be more physically active than the general population [43,44,45]. This may be caused by the fact that individuals who are already motivated to be physically active are more likely to participate in these types of interventions [46]. Moreover, in the specific case of this study, some participants became aware of the “Moving Parks” project through their sports associations and may have decided to participate, partly or entirely, because some activities were proposed by known instructors.

The results from the logistic regression analyses highlighted that higher age (+4%) and having a chronic health condition (+70%) were associated with a greater likelihood of participation in previous editions of the “Moving Parks” project. These results are promising, considering the beneficial effects of PA on elderly people and individuals with chronic diseases [1]. Many examples in the literature show that regular exercise may improve symptoms, functional capacity, and quality of life in individuals with chronic diseases (i.e., cardiovascular diseases, diabetes, and chronic pulmonary diseases) [47]. The benefits of PA for chronic health conditions are multifactorial, including effects on cardiovascular and respiratory function, skeletal muscle strength, insulin sensitivity, inflammation, and mental well-being [48,49]. Additionally, PA may have a positive effect on frailty prevention in elderly people [48]. This type of intervention should be encouraged by patients suffering from chronic diseases, and efforts should be made to maintain their continuous participation in the studied project.

While the analysis highlighted some significant predictors, such as higher age and the presence of a chronic health condition, it is also worth noting the reasonable but non-significant predictors, such as smoking habits. Even though the percentage of smokers among the participants was 14%, this factor did not significantly predict participation in the “Moving Parks” project. The lower prevalence of smoking among the participants as compared to the general population may suggest that non-smokers are more likely to participate in physical activity promotion interventions. Nevertheless, promoting physical activity among smokers can offer them a valuable tool for quitting, given that physical activity can help manage withdrawal symptoms and cravings, reduce negative mood and weight gain associated with quitting, and improve health outcomes [50].

Continuous participation in the project was associated with higher self-reported levels of PA (+42%) and meeting the recommended PA during the winter months (+42%). These results support the findings of previous studies on the effectiveness of the “Moving Parks” project in increasing participants’ levels of PA [30,31]. However, as discussed before, individuals that participate in this type of PA promotion intervention may be more active than the general population. Conceivably, the association between continuous participation and higher levels of PA should not be interpreted as conclusive but as supportive of the findings of the studies on previous editions of the project.

Users’ characteristics, such as age, gender, educational level, and having a chronic disease, were heterogeneously associated with different communication media in the study sample, suggesting that different strategies may be employed in order to promote the following editions of the project to reach specific targeted population subgroups.

As a possible further strategy, participants may be encouraged to disseminate information about the project to their social circle that has not been reached by leveraging social influence. This could lead to increased participation and engagement among the previously “hard-to-reach” population subgroups [51]. This strategy may be integrated into already existing peer-to-peer health promotion projects in the Bologna area, enhancing the reach and impact of the “Moving Parks” project and further advancing the goal of promoting PA and preventing non-communicable diseases.

### 4.2. Study Limitations

This study has some limitations that should be described and acknowledged. First, causal inferences may be limited due to the cross-sectional design of the study. Furthermore, the items of the survey used as variables in the data analyses were self-reported, exposing the study to biases that may affect the accuracy and reliability of the results. For example, participants may not accurately report their PA levels or their health condition, or they may be influenced by social desirability biases. Most importantly, the generalizability of the results may be compromised due to the existing differences between the sample and the general population in terms of gender, educational level, and PA level distributions.

## 5. Conclusions

This study provides an insight into the participants’ characteristics of a park-based PA promotion intervention. The results of the study showed that most of the citizens who participated in the 2022 edition of the “Moving Parks” project were women, highly educated, and already physically active. Moreover, continuous participation in the project appears to be more prevalent in individuals with a higher level of education, age, and presence of chronic diseases. Those findings suggest the presence of gender differences and education-based disparities in access to this health promotion intervention, as well as its effectiveness in appealing to more vulnerable subjects.

Further adjustments are needed in order to promote this intervention among the less affluent segments of the population. Since the results of this study highlighted how different population subgroups acknowledge the existence of the project through different media, a targeted strengthening of the advertisement campaign may reach those less represented segments of the population.

## Figures and Tables

**Table 1:** Sample characteristics.

Characteristics	n = 596 ^1^
**Gender**	
Female	503 (85%)
Male	91 (15%)
Missing	2
**Age**	49.74 ± 16.48
Missing	32
**Educational level**	
Lower than high school	61 (10%)
High school degree	201 (34%)
University degree	331 (56%)
Missing	3
**Smoking habit**	
No	511 (86%)
Yes	83 (14%)
Missing	2
**Presence of chronic conditions**	
No	463 (78%)
Yes	129 (22%)
Missing	4
**Media thanks to which participants became aware of the project**	
Sport association	66 (11%)
Internet	133 (23%)
Newsletter	31 (5.3%)
Walking in the park	26 (4.5%)
Word-of-mouth	201 (35%)
Social Media	88 (15%)
Television	1 (0.2%)
Billboard/Flyer	34 (5.9%)
Missing	16
**Reason of participation**	
”It is free”	67 (12%)
”The parks are easy to reach”	47 (8.1%)
”I appreciate the activities”	132 (23%)
”The project allows me to stay outdoors and in nature”	146 (25%)
”I feel good after participating”	147 (25%)
”I appreciate other participants presence”	13 (2.2%)
”A mix of the above reasons”	26 (4.5%)
Missing	18
**Continuous participation**	
No	284 (48%)
Yes	312 (52%)
**Reached recommended Physical Activity levels**	
No	306 (51%)
Yes	290 (49%)
**Physical Activity during winter months**	
No	158 (27%)
Yes	438 (73%)
**Moderate Physical Activity**	
No	198 (46%) *
Yes	237 (54%) *
**Vigorous Physical Activity**	
No	248 (59%) *
Yes	170 (41%) *

^1^ n (%); Mean ± SD. * Relative frequencies refer to the sub-sample of those active during the winter months.

**Table 2:** Predictors of continuous participation.

Predictors	Odds Ratios	95% CI	*p* ^1^
**Gender**			
Female	-	-	
Male	1.52 (+52%)	0.93–2.50	0.096
**Age**	1.04 (+4%)	1.03–1.05	**<0.001**
**Educational level**			
Lower than high school	-	-	
High school degree	2.01 (+101%)	1.03–3.92	**0.041**
University degree	2.05 (+105%)	1.04–4.06	**0.039**
**Smoking habit**			
No	-	-	
Yes	1.05 (+5%)	0.63–1.75	0.858
**Chronic conditions**			
No	-	-	
Yes	1.70 (+70%)	1.08–2.71	**0.023**

^1^ statistically significant *p* values are reported in bold format.

**Table 3:** Predictors of PA and recommended PA levels during the winter months.

Physical Activity			
Predictors	Odds Ratios	95% CI	*p* ^1^
**Continuous participation**			
No	-	-	
Yes	1.82 (+82%)	1.21–2.76	**0.004**
**Gender**			
Female	-	-	
Male	1.15 (+15%)	0.67–2.04	0.621
**Age**	1.02 (+2%)	1.00–1.03	**0.014**
**Educational level**			
Lower than high school	-	-	
High school degree	1.23 (+23%)	0.57–2.54	0.585
University degree	1.32 (+32%)	0.61–2.75	0.463
**Smoking habit**			
No	-	-	
Yes	0.82 8–18%)	0.48–1.43	0.470
**Chronic conditions**			
No	-	-	
Yes	0.93 (−7%)	0.56–1.57	0.777
**Recommended PA levels**			
Predictors	Odds Ratios	95% CI	*p*
**Continuous participation**			
No	-	-	
Yes	1.46 (+46%)	1.02–2.08	**0.038**
**Gender**			
Female	-	-	
Male	1.26 (+26%)	0.79–2.02	0.336
**Age**	1.00 (+0%)	0.99–1.01	0.741
**Educational level**			
Lower than high school	-	-	
High school degree	1.89 (+89%)	0.99–3.67	0.055
University degree	2.09 (+109%)	1.09–4.10	**0.029**
**Smoking habit**			
No	-	-	
Yes	1.13 (+13%)	0.69–1.86	0.629
**Chronic conditions**			
No	-	-	
Yes	1.09 (+9%)	0.71–1.68	0.693

^1^ statistically significant *p* values are reported in bold format.

## Data Availability

Data can be shared upon reasonable request.

## Supplementary Materials

The following supporting information can be downloaded at: https://www.mdpi.com/article/10.3390/healthcare11162287/s1. Table S1: Analyses of associations between dissemination media and users’ characteristics.
