# Supplementary material for: Participants Characteristics of a Park-Based Physical Activity Intervention in an Urban Context—A Cross-Sectional Study in Bologna, Italy
Source: Healthcare (Basel). 2023 Aug 14;11(16):2287. doi: 10.3390/healthcare11162287 (PMC10454791; doi:10.3390/healthcare11162287)
Supplement: Supplementary file 1 [file healthcare-11-02287-s001.zip › healthcare-2534850-supplementary.pdf]

Supplementary Material

Questionnaire - Translated version of the survey questions

1 - Gender ☐ M ☐ F ☐ Other
2 - Date of birth __________
3 - What is your educational background?

☐None
☐Elementary school degree
☐Middle school degree
☐High school degree
☐University degree

4 – How did you become aware about the "Moving Parks" project? (Select one answer)

☐ Billboard/flyer ☐ Walking in the park
☐ Through my sport association ☐ Word-of-mouth
☐ Social media ☐ Newsletter via e-mail ☐ Internet Websites
☐ Other

5 – Did you participate in the previous edition of "Moving parks"? ☐ No ☐ Yes

6 – Do you suffer from a chronic health condition? ☐ No ☐ Yes

7 – Are you a smoker? ☐ No ☐ Yes

8 – During the winter period, when "Moving parks" is not active, did you practice physical activity regularly?
☐ I did not practice physical activity regularly ☐ I practiced physical activity regularly

8.1 – If you responded "I practiced physical activity regularly", how many time did you spend practicing:

Moderate Physical Activity				
Working hard enough to raise your heart rate or respiratory rate and sweat.<br>(In example: brisk walking, cycling, dancing, gardening, cleaning the house)				
Less than 1 hour a week ☐	Around 1 hour a week ☐	Around 2 hours a week ☐	Around 2 hours and a half a week ☐	More than 2 hours and a half a week ☐

Attività fisica vigorosa

Working hard enough to raise a lot your heart rate, respiratory rate and having a high increase of sweat.<br>(In example: running, cycling rapidly, workout in a gym, aerobic sport training)				
Less than 30 min a week<br>☐	Around 30 min a week<br>☐	Around 1 hour a week<br>☐	Around 1 hour and 15 min a week<br>☐	More than 1 hour and 15 min a week<br>☐

9 – What is the reason that prompted you to participate in the "Moving parks" project?

☐ It's free
☐ The parks are easy to reach
☐ I appreciate the activities
☐ The project allows me to stay outdoor and in nature
☐ I feel good after participating
☐ I appreciate other participants presence

Table S1. Analyses of associations between dissemination media and users' characteristics.

Sport Association			
Predictors	*Odds Ratios*	*95% CI*	*p*
**Gender**			
Female	-	-	-
Male	1.11	0.52 – 2.18	0.781
**Age**	1.02	1.00 – 1.04	**0.027**
**Educational level**			
Lower than high school			
High school degree	1.86	0.70 – 5.92	0.245
University degree	1.63	0.59 – 5.36	0.381
**Smoking habit**			
No	-	-	-
Yes	1.32	0.57 – 2.78	0.486
**Chronic conditions**			
No	-	-	-
Yes	0.86	0.43 – 1.64	0.663
**PA during winter**			
No	-	-	-
Yes	3.35	1.51 – 8.91	**0.007**

Internet			
**Gender**			
Female	-	-	-
Male	1.05	0.59 – 1.79	0.871
**Age**	1.00	0.99 – 1.02	0.720
**Educational level**			
Lower than high school	-	-	-
High school degree	1.42	0.63 – 3.54	0.417
University degree	1.91	0.85 – 4.75	0.137
**Smoking habit**			
No	-	-	-
Yes	0.74	0.38 – 1.36	0.354
**Chronic conditions**			
No	-	-	-
Yes	1.14	0.67 – 1.88	0.624
**PA during winter**			
No	-	-	-
Yes	0.87	0.55 – 1.39	0.562
Newsletter			
**Gender**			
Female	-	-	-
Male	0.37	0.06 – 1.28	0.184
**Age**	1.03	1.00 – 1.06	0.074
**Educational level**			
Lower than high school			
High school degree	1.38	0.32 – 9.60	0.696
University degree	2.74	0.66 – 18.94	0.218
**Smoking habit**			
No	-	-	-
Yes	0.24	0.01 – 1.20	0.172
**Chronic conditions**			
No	-	-	-
Yes	1.07	0.40 – 2.59	0.882
**PA during winter**			
No	-	-	-
Yes	0.83	0.37 – 2.08	0.678
Word-of-mouth			

**Gender**			
Female	-	-	-
Male	1.34	0.83 – 2.17	0.227
**Age**	1.00	0.99 – 1.01	0.936
**Educational level**			
Lower than high school	-	-	-
High school degree	0.47	0.24 – 0.90	**0.023**
University degree	0.37	0.19 – 0.72	**0.003**
**Smoking habit**			
No	-	-	-
Yes	1.46	0.87 – 2.43	0.149
**Chronic conditions**			
No	-	-	-
Yes	0.91	0.57 – 1.44	0.685
**PA during winter**			
No	-	-	-
Yes	0.74	0.49 – 1.12	0.152
Social network			
**Gender**			
Female	-	-	-
Male	0.27	0.09 – 0.65	**0.007**
**Age**	0.97	0.95 – 0.98	**<0.001**
**Educational level**			
Lower than high school	-	-	-
High school degree	1.19	0.41 – 4.33	0.770
University degree	1.36	0.48 – 4.87	0.592
**Smoking habit**			
No	-	-	-
Yes	0.85	0.41 – 1.65	0.651
**Chronic conditions**			
No	-	-	-
Yes	0.72	0.34 – 1.41	0.361
**PA during winter**			
No	-	-	-
Yes	0.84	0.50 – 1.43	0.515
Billboard/flyer			
**Gender**			

Female	-	-	-
Male	3.53	1.54 – 7.79	**0.002**
**Age**	0.99	0.97 – 1.02	0.515
**Educational level**			
Lower than high school	-	-	-
High school degree	0.85	0.26 – 3.38	0.804
University degree	0.68	0.20 – 2.78	0.557
**Smoking habit**			
No	-	-	-
Yes	0.91	0.26 – 2.56	0.876
**Chronic conditions**			
No	-	-	-
Yes	3.39	1.48 – 7.71	**0.003**
**PA during winter**			
No	-	-	-
Yes	1.33	0.55 – 3.74	0.551
Walking in the park			
**Gender**			
Female	-	-	-
Male	0.57	0.09 – 2.06	0.460
**Age**	1.02	0.99 – 1.06	0.215
**Educational level**			
Lower than high school	-	-	-
High school degree	NA	NA	0.986
University degree	NA	NA	0.986
**Smoking habit**			
No	-	-	-
Yes	0.82	0.13 – 3.02	0.792
**Chronic conditions**			
No	-	-	-
Yes	0.32	0.05 – 1.21	0.144
**PA during winter**			
No	-	-	-
Yes	1.81	0.58 – 7.93	0.358
